# Neuron-Specific Enolase and S100B as Biomarkers of Ischemic Brain Injury During Surgery

**DOI:** 10.3390/clinpract15040074

**Published:** 2025-04-03

**Authors:** Matej Makovec, Milan Skitek, Leja Šimnovec, Aleš Jerin

**Affiliations:** 1 Department of Vascular Surgery, University Medical Centre Maribor, 2000 Maribor, Slovenia; 2 Institute of Clinical Chemistry and Biochemistry, University Medical Centre Ljubljana, 1525 Ljubljana, Slovenia; 3 Faculty of Pharmacy, University of Ljubljana, 1000 Ljubljana, Slovenia

**Keywords:** neuron-specific enolase, protein S100B, ischemic brain injury, surgery

## Abstract

Biochemical markers can be used in addition to neuroimaging techniques to evaluate the extent of ischemic brain injuries and to enable earlier diagnosis and faster intervention following the ischemic event. Among the potential biomarkers of ischemic brain injuries during surgery, neuron-specific enolase (NSE) and S100B are the most frequently studied and were shown to be the most promising. The aim of this review was to summarize the role of NSE and S100B as biomarkers of ischemic brain injuries that occur during selected surgical procedures, predominantly carotid endarterectomy (CEA). Some other invasive interventions that cause ischemic brain injuries, like extracorporeal membrane oxygenation, were also included. We can conclude that these biomarkers can be useful for the evaluation of ischemic brain injuries that occur during various surgical procedures. They can help to determine the most optimal conditions for performing the surgery and therefore improve the procedures to consequently minimize brain damage caused during surgery. Because of a significant delay between sample collection and obtaining the results, they are not suitable for real-time assessment of brain injuries. Some improvement can be expected with the future development of laboratory methods. The association of the changes in NSE and S100B levels during surgery with potential consequences of ischemic brain injury have been described in numerous studies. However, even in a very homogenous group of surgical procedures like CEA, these findings cannot be summarized into a common final conclusion; therefore, the prognostic value of the two markers is not clearly supported at the present time.

## 1. Introduction

Brain injuries are a common cause of morbidity and mortality worldwide. In general, brain injuries can be categorized into non-traumatic and traumatic injuries. Non-traumatic ischemic injuries can occur due to a lack of oxygen in the brain during some invasive surgical procedures and extracorporeal membrane oxygenation as well as after acute ischemic stroke and cardiac arrest [1]. Numerous studies aimed to identify specific biochemical markers that can indicate ischemic brain injuries in these conditions [2,3,4,5,6,7,8,9]. The release of these biomarkers can also be detected in other conditions not necessarily connected to ischemic injuries; biomarkers like neuron-specific enolase (NSE) and S100B can be elevated in infections and sepsis [10,11], and their increase can also be related to tumors [4,12]. Unlike non-traumatic injuries, which can have multiple internal causes, traumatic brain injuries are always caused by an external force; such injuries can be a consequence of falls, traffic accidents, sport-related accidents, pressure blasts, etc. [1]. In the past, traumatic brain injuries were considered as injuries with finite recovery, but today they are classified as chronic conditions [13]. The key to successful recovery from traumatic brain injury is early identification and treatment. Biochemical markers can be used in addition to neuroimaging techniques to evaluate the extent of an injury and allow for prognosis [5,14], especially in cases where neuroimaging techniques do not provide enough data.

Ischemic brain injuries are not just acute events but also involve delayed and progressive neurodegenerative processes with multiple interdependent cascades of biological reactions [15]. Therefore, one of the advantages of the biochemical markers of brain injury is the fact that they can correlate with different pathological processes.

In this review, we aimed to summarize the role of NSE and S100B as biomarkers of ischemic brain injuries during selected surgical procedures, predominantly carotid endarterectomy (CEA), and some other invasive interventions that can cause ischemic brain injuries, like extracorporeal membrane oxygenation.

## 2. Biomarkers of Brain Injury

The ideal biomarker for the diagnosis, prognosis, and monitoring of brain injury should be specific to the central nervous system (CNS), easily accessible, and resistant to cytoplasmic and extracellular proteolytic activity. The biomarker should have high diagnostic specificity and sensitivity and also some predicting value for both short-term and long-term outcomes. It is also important that the biomarker levels reflect the extent of CNS injury and improve with neuroprotective interventions [16].

Several candidates for biomarkers of brain injury have recently been studied, like markers of neuronal cell injury (NSE and ubiquitin C-terminal hydrolase L1), astroglial injury biomarkers (S100B and glial fibrillary acidic protein), αII-spectrin breakdown products [3], delayed axonal injury and demyelination markers (neurofilament proteins and myelin basic protein), postinjury neurodegeneration markers like Tau protein, and autoantibodies as biomarkers of autoimmune response. Other candidates include not only proteins like microtubule-associated protein 2, brain-derived neurotrophic factor, and postsynaptic protein neurogranin but also nucleic acids (microRNA and circulating nucleic acids), exosomes, and microvesicles [15,17,18]. Some other markers like clusterin [19], high-sensitivity troponin T [20], transmembrane protein-166 [21], brain natriuretic peptide [22], and visinin-like protein-1 [23] were used in an attempt to evaluate brain injury. Despite the confirmed correlations between the changes in biochemical markers and brain injuries, the use of individual markers is limited due to low sensitivity and specificity [24]. Among all the potential biomarkers of brain injury, NSE and S100B are the most studied and showed to be the most promising [25,26].

NSE is an isoenzyme of the enolase (EC 4.2.1.11). This cell-specific enzyme is an acidic soluble protein that was first described by Moore and McGregor in 1965 [27,28]. NSE is a homodimer composed of two γ subunits with a molecular mass of 78 kDa [27], which can be primarily found in the cytoplasm of neurons and is not normally secreted from cells [27,29]. It is released only in the event of neuronal damage, so it is considered to be a biomarker that directly assesses functional neuronal damage [27,29]. The half-life of NSE in serum is 24 h [16], and the serum concentrations typically rise within 6 h after injury [30]. Although NSE levels are highly specific for brain tissue, it is also expressed in other cell types like neuroendocrine cells, oligodendrocytes, platelets, and erythrocytes under certain physiological and pathological conditions [30,31].

The protein S100B belongs to a multigenic family of calcium-mediated proteins. Because of its calcium binding properties, S100B is involved in the regulation of various processes connected with the survival and proliferation of neuron cells [32]. The molecular mass of this homodimer cytosolic protein is 21 kDa [16], and its half-life was found to be between 0.5 and 2 h [26]. S100B is primarily found in astrocytes, but it is also present in other types of glial cells, like Schwann cells, oligodendrocytes, Müller cells, enteric glial cells, and ependymal cells. It can also be found in some non-neural cells, such as chondrocytes, melanocytes, adipocytes, dendritic cells of lymphoid organs, Langerhans cells, and certain types of lymphocytes [6]. Following cell injury, S100B is actively excreted in the extracellular space and also passively released from the cell [26]. In serum or cerebrospinal fluid, S100B is normally present in low concentrations, but its levels significantly increase in the event of intracranial injuries [30]. Additionally, S100B could be considered a biomarker of blood–brain barrier damage [16].

## 3. Ischemic Brain Injury During Surgery

The oxygen requirements of the brain are high, with minimal oxygen storage capacity. A significant reduction in the perfusion of oxygenated blood to the brain can lead to undesirable consequences in a very short time. Effective recognition of ischemia has been determined as a crucial element in reducing the risk of brain damage during surgery [33]. Since an event involving cerebral ischemia may not always result in an injury that could be diagnosed on the basis of conventional criteria, biomarkers can provide additional data to enable early diagnosis and timely intervention.

### 3.1. Carotid Endarterectomy

CEA is a surgical procedure for the removal of atherosclerotic plaques from the carotid arteries. Although the goal of this intervention is to minimize the long-term risk of ischemic stroke in individuals with severe carotid artery stenosis, one of the main complications of this surgery is exactly an ischemic stroke. The most frequent reason for this complication is reduced blood flow to the brain, which often occurs during cross-clamping of the internal carotid artery (ICA). The proper and immediate recognition of insufficient collateral flow is crucial for the successful performance of the surgery. During operation, a vascular shunt can be created, but this increases the complexity of the surgery and carries the risk of damage to the vascular wall, potentially leading to thromboembolism [34]. For this reason, good neuromonitoring is needed to identify patients who would benefit from the use of a vascular shunt. CEA can be performed under local or general anesthesia. Patients who receive local anesthesia are monitored clinically, which allows for more appropriate use of vascular shunts compared to other less sensitive neuromonitoring methods. Those who undergo surgery under general anesthesia cannot be observed clinically, so alternative successful neuromonitoring methods are sought for them [34,35,36].

Classical techniques used to monitor brain injuries during CEA include monitoring of carotid artery pressure, electroencephalography, transcranial Doppler sonography, somatosensory evoked potentials, near-infrared spectroscopy, and motor evoked potentials. Although these methods are effective and routinely used, some can be invasive. Specific biomarkers of brain injury have been studied as a good alternative [37,38] since even small cerebral injuries after surgery can be associated with significant increases in serum biomarkers like S100B [39].

Dragas [25] found that the concentration of NSE decreased after declamping and 24 h following conventional CEA using a shunt and Dacron patch, while the NSE levels slightly increased after eversion CEA without the use of a shunt. The study demonstrated that NSE concentrations seem to be affected by the use of shunt. Routine shunting during CEA could therefore prevent an increase in serum NSE concentration. In this study, the method of CEA, whether using a shunt or not, had no effect on S100B levels, indicating different mechanisms of this marker’s release. In both cases, the S100B levels were elevated after declamping and then began to decrease 24 h after the operation.

Kuzhuget [37] studied how stump pressure and cerebral oximetry (rSO2) values influence the assessment of ischemic brain injury during CEA without shunting. During the operation, stump pressure, rSO2, ΔrSO2 (↓rSO2 from baseline), and the biomarkers NSE and S100B were measured. He found that clamping time affects S100B levels, meaning that, the longer the clamping time, the higher the level of this protein. Monitoring S100B levels at five time-points showed that S100B levels rise during surgery, return to the baseline on the first day after surgery, and then drop under the baseline on the following day. Similar to S100B, NSE also increased, but the levels remained elevated slightly longer, returning to the baseline only on the third day. Although the NSE and S100B levels changed over time, there was no observable influence of stump pressure, rSO2, and ΔrSO2 parameters on the NSE and S100B levels. Kuzhuget concluded that neither stump pressure nor cerebral oximetry are effective predictors of ischemic brain injury.

Wijeyaratne [40] studied the difference between local and general anesthesia during CEA on the extent of brain injury. He demonstrated that jugular venous NSE levels significantly increase after CEA under general anesthesia compared to local anesthesia. This suggests that CEA performed under local anesthesia preserves perfusion and cerebral oxygenation better compared to CEA under general anesthesia, thereby providing some protection against perioperative brain injury. General anesthesia was shown to cause an elevation of NSE in elderly patients [7]; the increase in NSE was correlated with the severity of postoperative cognitive dysfunction.

Iłżecki [29] measured the serum NSE levels in patients who underwent CEA under local anesthesia without the use of a shunt. This study showed that the timing of sampling greatly affects serum NSE levels, with a statistically significant increase 48 h after CEA compared to the levels before surgery and also 12 h postoperatively. There was also no significant correlation between serum NSE levels and clamping time of internal carotid artery during CEA. Additionally, no statistically significant connection was found between the serum NSE levels in symptomatic and asymptomatic patients, nor between the NSE levels and blood flow velocity in the internal carotid artery before and after CEA. Although the precise mechanism of increased NSE in this study is unknown, the researchers suggest that hypoxia during CEA might increase the permeability of the blood–brain barrier, which consequently leads to NSE leakage into the blood.

In our recent study [9], we examined the relationship between neurological instability in patients post-CEA and elevated serum levels of S100B, along with decreased rSO2 levels. The study revealed that awake neuromonitoring enables direct assessment of cerebral tissue perfusion and is specific for CEA performed under local anesthesia. One of the main findings of this study was that an increase in serum S100B correlates with neurological instability. The increase in S100B predicted neurologic change with a sensitivity and specificity of 71.4% and 75.4%, respectively. However, due to the time difference between sample collection and obtaining results (usually about 3 h), real-time monitoring of serum S100B during surgery was not possible. Similar to the study performed by Kuzhuget [37], no significant correlation was found between rSO2 decline and neurological symptoms.

In spite of numerous studies that have examined the association of the changes in NSE and S100B levels with various states of ischemic brain injury during CEA, these findings cannot be summarized into a common final conclusion. While some researchers were able to establish a connection between elevated NSE and S100B levels and brain injuries in patients who underwent CEA [9,25,29,37,39,40], others failed to confirm this correlation [41,42,43]. The differences might be partly caused by various states of ischemic brain injury included in these studies.

### 3.2. Cardiac Surgery

In cardiac surgery, particularly during the procedures involving cardiopulmonary bypass, the brain is susceptible to ischemic injury. The perioperative period is another critical part as hypoxic–ischemic injuries can happen during this time [44]. Potential biomarkers like NSE and S100B have been evaluated for the detection of early-stage perioperative brain injuries following cardiac surgery. These biomarkers are thought to be associated with acute brain injury after cardiac surgery, but their ability to predict postoperative cognitive dysfunction following cardiac surgery remains uncertain.

Several previous studies found increases in NSE and S100B during cardiac surgery. While S100B in serum peaked after cardiopulmonary bypass, this event has not significantly influenced the serum levels of NSE [45]. A correlation between elevated levels of these biomarkers and adverse neurological outcomes was also indicated. However, some recent studies [46], which focused on postoperative cognitive dysfunction, found no significant correlation with NSE up to 48 h after surgery. Even in studies with longer follow-up (up to five days), NSE did not prove to be an independent predictor for poor outcomes, while S100B measured on the first postoperative day was anticipated to predict some adverse neurological outcomes with high specificity [47]. Although S100B seems to be the more promising of both mentioned biomarkers, it was confirmed that it has several limitations, predominantly due to the potential release from non-neural sources [44], especially in pediatric populations.

The reliability of NSE measurement can additionally be limited by pre-analytical factors. Even mild hemolysis, which may arise during cardiopulmonary bypass, can significantly elevate serum NSE levels [48], making it challenging to differentiate between neural and non-neural sources of this enzyme.

## 4. Other Interventions and Conditions Causing Ischemic Brain Injury

Acute ischemic brain injury can occur during extracorporeal membrane oxygenation (ECMO). Therapies like ECMO can provide very beneficial support for treating patients with severe respiratory or cardiac failure, but, on the other hand, ECMO carries a significant risk of morbidity or mortality due to acute brain injuries [2,8]. Patients undergoing ECMO are often deeply sedated, and neuromonitoring can be highly invasive, so obtaining a comprehensive neurological assessment of these patients is quite challenging. For this reason, many studies focused on identifying the connection between the serum levels of biomarkers like S100B and intracranial lesion development. Fletcher-Sandersjöö et al. [2] found that elevated serum S100B levels were associated with the development of intracranial lesions during ECMO treatment. The best prediction of lesions was at sampling times between 40 and 140 h after the start of ECMO. Reuter et al. [49] studied patients treated with ECMO after refractory cardiogenic shock or cardiac arrest. They determined that an elevation of serum NSE on the third day could predict poor functional outcomes and mortality.

Elevated levels of NSE and S100B can also be measured in serum to evaluate brain injury after acute ischemic stroke. Although NSE and S100B have been considered as promising biomarkers and have been extensively studied, it has not been proven that they significantly contribute to improved stroke diagnosis [50,51]. The diagnostic and prognostic value might be improved by combining them in panels and by performing repeated measurements. Combining NSE with S100B can also improve the accuracy of brain injury prognosis after cardiac arrest. While S100B can peak earlier (around 24 h), NSE may provide a better indication of long-term neurological outcomes, with the highest prognostic accuracy 48 h after the event [43].

## 5. Conclusions

Biomarkers like NSE and S100 can be useful for the evaluation of ischemic brain injuries that occur during various surgical procedures. They can help to determine the most optimal conditions for performing surgery, and this optimization could consequently improve the procedures and minimize brain damage during surgery. A major drawback is a significant time delay between sample collection and obtaining the results, so these biomarkers are not suitable for real-time assessment of brain injuries during surgery. Some improvement can be expected with further development of the laboratory methods.

The association of the changes in NSE and S100B levels during surgery with potential consequences of ischemic brain injury have been described in numerous studies. These findings cannot be summarized into a common final conclusion; therefore, the prognostic value of the two markers is not clearly supported at the present time.

## Data Availability

No new data were created or analyzed in this study. Data sharing is not applicable to this article.
